# The Use of the Self-Standing Turning Transfer Device to Perform Bed-To-Chair Transfers Reduces Physical Stress among Caregivers of Older Patients in a Middle-Income Developing Country

**DOI:** 10.3389/fmed.2014.00032

**Published:** 2014-09-25

**Authors:** Choon Hian Goh, Muslimah Y, Siew-Cheok Ng, Pathmawathi Subramanian, Maw Pin Tan

**Affiliations:** ^1^Department of Biomedical Engineering, Faculty of Engineering, University of Malaya, Kuala Lumpur, Malaysia; ^2^Institute for Public Health, Kuala Lumpur, Malaysia; ^3^Department of Nursing Studies, Faculty of Medicine, University of Malaya, Kuala Lumpur, Malaysia; ^4^Ageing and Age-Associated Disorders Research Group, Faculty of Medicine, University of Malaya, Kuala Lumpur, Malaysia; ^5^Division of Geriatric Medicine, Department of Medicine, Faculty of Medicine, University of Malaya, Kuala Lumpur, Malaysia

**Keywords:** aged, carer, transfer devices, early rehabilitation, back injury

## Abstract

Manual transfer of elderly patients remains commonplace in many developing countries because the use of lifting equipment, such as hoists, is often considered unaffordable luxuries. The aim of this study was, therefore, to evaluate the usage and potential benefits of a low-cost, mechanical turning transfer device among elderly patients and their caregivers on a geriatric ward in a developing country in South East Asia. Fifty-six inpatients, aged 66–92 years, on a geriatric ward, and their caregivers were recruited. Participants were asked to transfer from bed-to-chair transfer with manual assistance, and the task was repeated using the Self-standing Turning Transfer Device (STurDi). The time taken to perform manual transfers and STurDi-assisted transfers was recorded. Physical strain was assessed using the perceived physical stress-rating tool for caregivers with and without the use of the device. User satisfaction was evaluated using the usefulness, satisfaction, and ease of use questionnaire. There was a significant reduction in transfer-time with manual transfers compared to STurDi-assisted transfers [mean (SD) = 48.39 (13.98) vs. 36.23 (10.96); *p* ≤ 0.001]. The physical stress rating was significantly lower in STurDi-aided transfers compared to manual transfers, shoulder [median (interquartile range) = 0 (1) vs. 4 (3); *p* = 0.001], upper back [0 (0) vs. 5 (4); *p* = 0.001], lower back [0 (1) vs. 5 (3), *p* = 0.001], whole body [1 (2) vs. 4 (3), *p* = 0.001], and knee [0 (1) vs. 1 (4), *p* = 0.001]. In addition, majority of patients and caregivers definitely or strongly agreed that the device was useful, saved time, and was easy to use. We have therefore demonstrated in a setting where manual handling was commonly performed that a low-cost mechanical transfer device reduced caregiver strain and was well received by older patients and caregivers.

## Introduction

Majority of older persons in the world now reside in developing countries (1). Functional impairment among older persons in these settings is more common than that reported in the published literature from high-income countries (2). Mobility limitations in frail elderly patients are often dynamic and characterized by frequent transitions between states of independence and disability (3). Older individuals, therefore, often require assistance to perform physical tasks such as transferring from bed to chair. Manual lifting is often the only available method to the caregiver to perform lifting and transfer tasks for older patients (4), despite the fact that manual handling is one of the major causes of back injuries among caregivers (5).

Mechanical and electrical devices are not often used to assist with the lifting and transfer tasks of older patients due to limited availability of such equipment as well as the reluctance of caregivers to use lifting and transfer aids. The availability of assistive devices is often limited by the high cost of commercially available devices relative to the average income of the population. In addition, the appropriate use of lifting and transfer aids is also limited by the lack of available training, which is also often not available (6). Low-cost, easy-to-use equipment is, therefore, much needed in lower to middle-income countries where the aging population is increasing at an accelerated rate.

In response to the need for affordable and easy to operate assistive devices in such setting, the researchers have developed a simple, mechanical device to assist with standing transfer maneuvers among our older patients. The researchers compared the use of manual transfers and transfers with assistance from a mechanical device in order to determine the potential differences in time required to perform the transfer maneuver, caregiver’s physical stress, and the overall acceptability of the device by caregivers and patients.

## Materials and Methods

### Participants

Consecutive older inpatients admitted to the acute geriatric ward at the University of Malaya Medical Centre (UMMC), Kuala Lumpur, Malaysia, over a 10-week period were screened for suitability for the study. The UMMC is a large 1,000-bedded teaching hospital, which also serves as a general hospital for its local catchment population of 300,000 at the western border of the capital city of Kuala Lumpur and its adjacent city of Petaling Jaya. The acute geriatric ward at the UMMC consists of 30 beds with 70–100 admissions per month. The inclusion criteria were an elderly mobility score (EMS) of <10, requiring assistance of at least one person to perform transfer maneuvers, adequate upper limb strength to participate in transfers, able to obey simple commands, able to cooperate with transfer maneuvers, and the presence of at least one informal or paid caregiver to assist in the maneuver. Participants were excluded if they were able to perform bed-to-chair transfers independently, if they were unable to stand with the maximal assistance of two persons, if they were unable to provide informed consent, if they did not have a caregiver available, or if their caregiver declined participation in the study. Bed-to-chair transfers were defined as moving from the seated position by the side of the bed to the standing position, pivoting the body by at least 90° before sitting down on a chair or wheelchair.

### Ethical approval and informed consent

This study had been approved by the UMMC Medical Ethics Committee [1030.16 (1)]. Written informed consent was obtained from both participants and caregivers.

### Self-standing Turning Transfer Device

**Figure 1:**
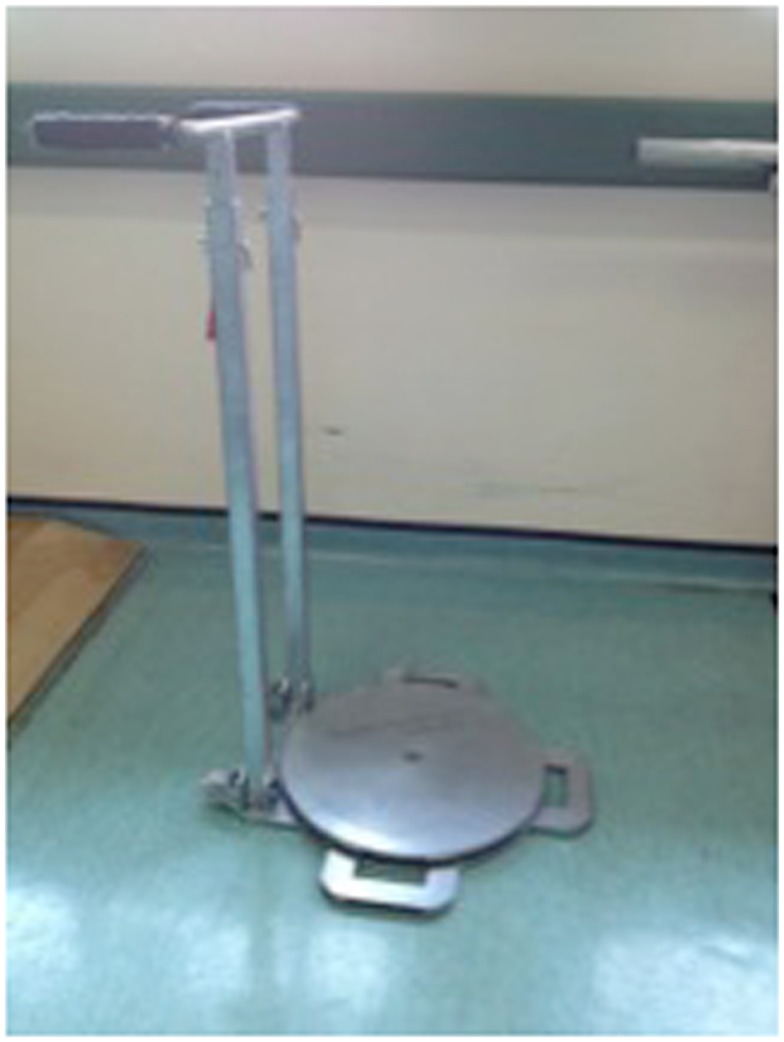
**The self-standing turning transfer device**. The frame is made of mild steel. The base is stabilized by the weight of the device and its two small feet. The rotating disk turns easily on the base. The handle is height adjustable.

The self-standing turning transfer device (STurDi) (patent pending) is a mechanically operated device, constructed from mild steel, developed by biomedical engineers at University of Malaya. A patent application has been filed for this device. The base consists of a disk-shaped turning footplate on a wider steel base, with minimal elevation from ground level. The handle can be adjusted to the appropriate height, and is connected to the base via two thick steel bars (Figure 1). The device had been pre-tested by normal healthy volunteers and was found to be able to withstand weights of up to 200 kg without warping of the steel frame or reduction in the turning function. The bearing used for the device was obtained from Luoyang JiaWei Bearing Manufacturing Co. Ltd., which has implemented the ISO 9001–2008 quality management system. The diameter standard of the bearing was GB/T4663-1994 and the tolerance standard was GB/T 307.42-2002. The mild steel construction had minimal yield strength of 250 MPa (AK Steel Corporation, 2007) and the minimum yield strength of the bolts and nuts used was 393 MPa (K-T Bolt Manufacturing, Inc., 2005).

### Study design

#### Manual transfers

Participating patients were first instructed to perform manual standing transfers from bed-to-chair using safe manual transferring techniques. This would include the maximal assistance of two caregivers if this was normally required. At least one of the caregivers assisting with the standing transfer maneuvers would be the patient’s regular caregiver, who may either be a family member or a paid caregiver (usually a foreign domestic worker). The time taken to perform this manual standing transfer task (transfer-time) was recorded from the time when the patient’s bottom leaves the mattress to the point at which the patient’s back touches the back of the chair.

#### STurDi-assisted transfers

The same task was then repeated using the STurDi device, with the original pair of caregivers who assisted with the manual standing transfer. The caregivers were instructed to allow the patient to perform as much as possible the transfer maneuver by himself or herself, observe closely and provide adequate assistance (if required) to ensure a safe transfer. The patients were first verbally instructed on how to use the device using pictures as a visual aid. They were told to position themselves to a sitting position, with or without assistance, on to the side of the bed. With their feet positioned slightly apart on the footplate, patients were then told to pull themselves up to a standing position using the handle bar. Patients would have to initiate the turning action themselves by moving their hands across the handle bars so that their back would turn toward the direction of the chair, before sitting down on the chair or wheelchair of appropriate height. The caregiver would have his or her foot positioned at the edge of the footplate, and apply foot pressure on the footplate if necessary to prevent the turning disk from turning too quickly or over spinning. The patients and their caregivers were observed using the STurDi device to first transfer from the bed to a chair, and from the chair back to the bed in the trial run, before performing the actual timed STurDi-aided bed-to-chair transfer. The timing of only one transfer maneuver from bed to chair was measured and recorded.

### Data collection

The patient’s age, gender, and ethnicity were obtained while the caregiver’s age, gender, and level of education were recorded. Limitation in lower limb function among the participating patients was assessed with the EMS. The total score for the EMS is 20 with a score of <10 indicating dependence in mobility maneuvers and requiring help with basic ADLs. The physical stress-rating (7) index was obtained from the caregivers following the manual transfer procedure and after using the STurDi device. The physical stress-rating index consisted of 5 items, which measured physical strain on a Likert scale of 0–9, with 0 indicating no strain and 9 indicating maximal strain. The score for each item as well as the total score was compared. The usefulness, satisfaction, and ease of use (USE) questionnaire, which was developed for the purpose of evaluating software interface usability was adopted to determine usability among caregivers and patients in the study (8). It is a 30-item questionnaire, which evaluated usability within the 4 domains of usefulness, satisfaction, ease of learning, and ease of use on a seven-point Likert scale with 1 indicating strongly disagree and 7 indicating strongly agree. The domain score and the total score were calculated as percentages to determine degrees of usefulness, satisfaction, ease of learning, and ease of use, as well as total USE score.

### Data analysis

Normal distributions were determined for continuous data using histograms and the Komolgorov–Smirnov test. Normally distributed continuous variables were presented as means with SD in parenthesis while non-normally distributed variables were presented as medians with interquartile ranges. Categorical variables were presented as frequencies with percentages in parentheses. Paired comparisons were made for the transfer-time as well as physical stress reported by carers for manual and STurDi-assisted transfers using the paired sample *t*-test and the Wilcoxon signed-rank test, respectively.

## Results

### Demographic characteristics of patients and caregivers

One hundred and fifty-six patients were admitted into the acute geriatric ward from 17 January 2014 until 30 April 2014. Sixty-three of the 156 patients (40%) fulfilled the selection criteria. Out of 63 potential participants, 3 caregivers and 4 patients refused to participate. Fifty-six of the 63 elderly patients (89%) and their caregivers were therefore enrolled into the study. The age range and demographic characteristics of patients and caregivers and EMS scores of patients are summarized in Table 1. Forty-five (80%) caregivers were related to the patients while 11 (20%) were paid caregivers. One (2%) caregiver had no formal education, 2 (4%) had only primary school education, 33 (59%) had secondary education, and 20 (36%) had tertiary education. The average monthly household income was less than the national average of RM5,000 for 30 (54%) of patients, while the remaining 26 (46%) reported an average household income of greater than RM5,000.

**Table 1:** **Demographic data and elderly mobility scores**.

Variable	Patient (*n* = 56)	Caregivers (*n* = 56)
Mean age (range), years	79.1 (66–92)	35.3 (19– 61)
Gender, *n* (%)
Female	37 (66)	40 (71)
Ethnicity, *n* (%)
Malay	21 (37)	20 (36)
Chinese	23 (41)	18 (32)
Indian	11 (20)	11 (20)
Others	1 (2)	7 (13)
Elderly mobility scale, *n* (%)
Lying to sitting
Independent	3 (5)	
Needs help of 1 person	51 (91)	
Needs help of 2+ people	2 (4)	
Sitting to lying
Independent	3 (5)	
Needs help of 1 person	50 (89)	
Needs help of 2+ people	3 (5)	
Sit to stand
Independent in under 3 s	0 (0)	
Independent in over 3 s	1 (2)	
Needs help of 1 person (verbal or physical)	53 (94)	
Needs help of 2+ people	2 (4)	
Standing
Stands without support and reaches	0 (0)	
Stands without support but needs help to reach	7 (12)	
Stands, but requires support with upper limbs	45 (80)	
Stands, only with physical support (1 person)	4 (7)	
Gait
Independent	1 (2)	
Independent with frame	7 (12)	
Mobile with walking aid but erratic/unsafe turning	33 (59)	
Requires physical assistance or constant supervision	15 (27)	
Timed walk
Under 15 s	3 (5)	
16–30 s	4 (7)	
Over 30 s	49 (88)	
Functional reach
Over 20 cm	0 (0)	
10–20 cm	17 (30)	
Under 10 cm or unable	39 (70)	

### Transfer-time

Table 2 summarizes the mean transfer-time for both manual transfers and STurDi-assisted transfers. There was a significant reduction in mean time required to perform bed-to-chair transfers using STurDi-assisted transfers compared to manual transfers (*p* < 0.001).

**Table 2:** **Mean transfer-time for manual transfers and STurDi-assisted transfers**.

Variable	Mean (SD)		Mean difference (95% CI)	*T* statistic (df)	*p*-Value*
	Manual transfers	STurDi transfers	
Time (s)	48.39 (13.98)	36.23 (10.96)	12.16 (10.35–13.97)	13.49 (55)	<0.001

*SD, standard deviation*.

*Paired *t*-test.

### Physical stress rating for caregivers

The median (interquartile range) for the individual components of the physical stress-rating scale completed by caregivers following routine manual transfers and STurDi-assisted transfers are compared in Table 3. There were significant differences in the median scores for individual component scores and total physical stress-rating scores between manual transfers and STurDi-assisted transfers (*p* < 0.001).

**Table 3:** **Physical stress-rating scores for manual and STurDi transfers**.

	Manual transfer	Using the self-standing turning transfer device	*p*-Value*
Shoulder median (IQR)	4 (3)	0 (1)	<0.001
Upper back median (IQR)	5 (4)	0 (0)	<0.001
Lower back median (IQR)	5 (3)	0 (1)	<0.001
Whole body median (IQR)	5 (4)	1 (2)	<0.001
Knee median (IQR)	1 (4)	0 (0)	<0.001

*Wilcoxon signed-rank test.

*IQR, interquartile range*.

### USE questionnaire scores

The Cronbach’s alpha for patients and caregivers for the USE questionnaire was 0.872 and 0.965, respectively. The mean usefulness, ease of use, ease of learning, and satisfaction scores for caregivers and patients are summarized in Table 4.

**Table 4:** **USE questionnaire scores for caregivers and patients**.

Item	Caregivers (*n* = 56)	Patients (*n* = 56)
Usefulness (%), mean (SD)	78 (16)	75 (17)
Ease of use (%), mean (SD)	74 (16)	71 (15)
Ease of learning (%), mean (SD)	87 (18)	87 (18)
Satisfaction (%), mean (SD)	75 (18)	72 (18)

*SD, standard deviation*.

## Discussion

This pragmatic, short-term evaluation of a mechanical device, which assists with standing transfers for individuals with lower limb weakness, had demonstrated that the STurDi device reduces time required to perform bed-to-chair transfers. It also reduces the physical stress rating among caregivers. In addition, both patients and caregivers found the STurDi device easy to use and were satisfied with it. Despite there being numerous published surveys and industry records of compensation claims, there are few published studies on nurses’, caregivers’, and patients’ experience of using assistive devices for lifting and transferring (9). Lifting and transfer devices are now widely used in geriatric care in high-income countries with “no lifting” policies commonly found in health care settings in the developed world. In lower to middle-income countries like Malaysia, however, assistive devices to help with lifting and handling are often considered unaffordable luxuries. In settings where lifting hoists are available, caregivers and healthcare professionals are reluctant to use it and still continue to transfer patients manually (10). The study has demonstrated that in a middle-income developing country, the introduction of a simple mechanical device to reduce the need for manual handling is acceptable to patients and caregivers.

The task of performing a bed-to-chair transfer requires the patient to stand from a seated position, rotate his or her body so that his or her back faces the new intended destination, and then to lower himself or herself on to the chair or wheelchair. When the maneuver is performed manually, the patient has to rise from the seated position assisted by the caregiver. At this position, the patient’s weight is partially supported by the caregiver. The pivoting maneuver that follows is often considered the most strenuous part of the transfer maneuver due to the twisting action on the caregiver’s back in order to support the patient to perform the pivoting maneuver. The stepping action to face another direction requires one foot to be lifted off the ground, potentially compromising the patient’s stability. The patient therefore compensates by taking multiple small steps to minimize the time spent on one leg. This delicate weight shifting process may also be unachievable in patients with inadequate lower limb strength, and in those who lack confidence in their ability. Depending on the patient’s physical ability, the standing transfer process often requires the help of more than one caregiver. The STurDi device is able to promote early mobility and independence by helping patients to achieve standing transfers earlier in their rehabilitation process and enhancing patient participation during standing transfers. As the older person is able to keep both feet firmly on the footplate throughout the transfer process, it increases the individuals’ level of confidence and also enables those who could not perform the pivoting maneuver to at least be able to perform the standing transfers. It also utilizes the patient’s upper limb strength in the transfer maneuver. This provides patients with better control of the maneuver, as they are able to initiate the transfer themselves rather than rely on the caregiver’s help.

Achieving standing transfers is often considered one of the first goals of rehabilitation after an acute illness. Skeletal muscle strength declines by 1–1.5% per day with strict bed rest (11) and 4–5% for each week of bed rest (12), which leads to a 10% reduction in postural muscle strength after 1 week of complete bed rest (13). Furthermore, elderly hospitalized patients spend most of their hospital stay in bed, despite their ability to walk independently (14). The promotion of early rehabilitation can be further enhanced with the use of the STurDi device and this is vital in preventing physical deconditioning, maintaining muscle function, and core stability, as well as regaining physical strength after an illness or injury.

Manual handling has been shown to be a cause of injury to the lower back by many authors, and exposure to the risk is pronounced in the health care industry (15). There was a significant reduction in physical stress burden with STurDi-assisted transfers compared to manual transfers. The study has therefore inferred that the use of the STurDi device is likely to reduce the risk of back injury through reduced physical stress. In addition, as the patient is also able to participate in the transfer process, the load of transfer is reduced and hence the number of caregivers required to perform the transfer maneuvers may also be reduced. The time required to perform the transfer maneuver was also significantly reduced. The STurDi device therefore reduces the caregiver’s workload. While this study was conducted with patients’ primary caregivers, in hospital wards, staff nurses would usually perform the transfer maneuver. Particularly in geriatric wards, where patients are more dependent, any interventions that could reduce staff time would be welcome. As the nurses are likely to perform several transfers on each patient each day, in a geriatric ward with 30 beds for instance, this will total hundreds of transfers per ward per day. The time saved in the transfer of patients will therefore amount to tangible staff cost savings. The STurDi device may also have a potential role in reducing healthcare professionals’ workload in busy geriatric and rehabilitation wards in developing countries.

In the search for existing devices with similar functionality in the world wide market yielded several devices that were not registered products in Malaysia. The features of three existing devices, the Patient Transfer (16), the Revo™ patient turner (Healthcraft, Canada), and the Rotunda™ transfer platform (Hawthorn Works, UK) were compared with the STurDi device. The Patient Transfer device comprises a seat, torso support, connector, and a rotary disk. A tiltable support can be employed by the caregiver to move the patient to and from the transport position of the patient supported by the rotary disk. However, the bulky steel frame of the design may cause difficulty with storage, and the transfer procedure is rather complicated, which limits its usability in developing countries. The Revo device comprises height adjustable handlebars, height and weight adjustable kneepads, and a rotating disk with locking mechanism. The drawback of this design is that it requires the assistance of the caregiver to lock and unlock the rotating disk. The Rotunda transfer platform comprises a *U*-shaped support and a bearing, which can be swiveled at five different positions. It is, however, rather bulky leading to storage difficulties. The STurDi device has comparable functionality, but was simpler to use and less bulky than the above devices. The retail price of the Patient Transfer is unknown, while the Revo and the Rotunda currently retails at around USD 625–1,000 according to market research. These prices do not include shipping costs and import duties. It is estimated that the manufacture and retail of the STurDi devices could be priced below RM1,000 (USD 300). Bed-to-chair patient transfers could also be performed using a manual sling, a rotating disk, or an overhead-lifting device. While the manual sling improves safety aspects, the requirement for the caregiver to lift the patient manually is still present, and hence does not alleviate the physical stress to caregivers. The transfer disk is highly portable and lower priced than other devices, but the lack of handles and stability reduces its functionality. Overhead-lifting devices such as transfer hoists are expensive, bulky, time-consuming to use, and often involve complicated steps that require training. While overhead-lifting devices are considered the safest option as it removes the need for lifting patients they are likely to promote early rehabilitation as patients are entirely passive during the transfer maneuver.

Patients and caregivers recorded high scores for all four domains of the USE questionnaire indicating the STurDi device has good usability. However, when asked whether they would buy the STurDi device, 55% of the patients and 45% of the caregivers strongly disagreed. The cost of both health and social care for older adults in Malaysia is mainly borne by out-of-pocket payments from family members. As a result, there is often little expendable cash to obtain what is perceived as optional luxuries. Therefore, while the STurDi device has demonstrated good usability in the healthcare setting, further evaluation will be required to assess the potential benefits and cost implications of providing this device in the community. While the cost of the device can be minimized by sourcing locally produced raw materials and local manufacturers, caregivers, and patients do appear unwilling or unable to spend any money on such equipment. The reasons behind this reluctance will need to be explored further.

The study was a cross-sectional study employing mainly surrogate participant reported outcomes. In order to clearly demonstrate that replacing manual handling with a mechanical device assisted process is effective in reducing caregiver workload, a much, larger prospective, randomized study measuring back injuries and work absence will be required. Such a study should ideally be conducted in the community where patients and their community caregivers could be provided with STurDi devices in their own homes or care institutions. If effective, the STurDi device should be made available in hospital wards, institutional care settings, and private homes in lower income countries where resources for equipment are even more limited.

## Conclusion

The use of the STurDi device is associated with significant reductions in physical stress of caregivers and transfer-time compared to manual transfers. Majority of the patients and caregivers definitely or strongly agreed that the device was useful, saved time, and was easy to use. This study has contributed to the sparse literature on the use of a lifting and transfer aid among caregivers worldwide. The STurDi device is potentially an affordable solution to reducing manual handling and preventing back injuries among caregivers in low- to middle-income countries. Further exploration of the use of the STurDi device in home settings is now indicated.

## Conflict of Interest Statement

The authors declare that the research was conducted in the absence of any commercial or financial relationships that could be construed as a potential conflict of interest.
